# Draft Genome Sequences of Six Strains of *Vibrio parahaemolyticus* Isolated from Early Mortality Syndrome/Acute Hepatopancreatic Necrosis Disease Shrimp in Thailand

**DOI:** 10.1128/genomeA.00221-14

**Published:** 2014-04-10

**Authors:** Hidehiro Kondo, Sasiwipa Tinwongger, Porranee Proespraiwong, Rapeepat Mavichak, Sasimanas Unajak, Reiko Nozaki, Ikuo Hirono

**Affiliations:** a Laboratory of Genome Science, Tokyo University of Marine Science and Technology, Tokyo, Japan; b Marine Shrimp Culture Research and Development Institute, Coastal Fisheries Research and Development Bureau, Department of Fisheries, Kasetklang Chatuchak, Bangkok, Thailand; c Charoen Pokphand Foods Public Co., Ltd., Aquatic Animal Health Research Center, Samutsakorn, Thailand; d Department of Biochemistry, Faculty of Science, Kasetsart University, Bangkok, Thailand

## Abstract

Some strains of *Vibrio parahaemolyticus* cause acute hepatopancreatic necrosis disease (AHPND) in shrimp. We sequenced 3 AHPND and 3 non-AHPND strains and found that all of them lacked the pathogenicity island relevant to human infection. A unique sequence encoding a type IV pilus/type IV secretion system was found in 3 AHPND strains.

## GENOME ANNOUNCEMENT

The production of cultured shrimp in several countries is being seriously impacted by early mortality syndrome (EMS), also called acute hepatopancreatic necrosis disease (AHPND) (1, 2). The causative agent of AHPND is *Vibrio parahaemolyticus* (2). However, in shrimp farms and elsewhere, not all strains of *V. parahaemolyticus* cause EMS/AHPND. A rapid and accurate diagnostic method is necessary for the control EMS/AHPND. To distinguish the pathogenic strains, we obtained the draft genome sequences of six strains of *V. parahaemolyticus*, which were isolated from different regions of Thailand: 3 AHPND strains from diseased shrimps (*Litopenaeus vannamei*) (TUMSAT_DE1_S1, TUMSAT_DE2_S2, and TUMSAT_D06_S3) and 3 non-AHPND strains from different shrimp farms (TUMSAT_H01_S4, TUMSAT_H03_S5, and TUMSAT_H10_S6). All AHPND strains were evaluated for their pathogenicity in living shrimps. All shrimps showed symptoms of AHPND similar to those observed in natural infection.

Bacterial DNA samples were prepared according to the method of Sambrook and Russell (3). The mate-pair libraries were generated from DNA using the Illumina Nextera XT DNA sample preparation kit. The libraries were sequenced using the Illumina MiSeq and the MiSeq reagent kits version 2 (300 cycles). The sequence data were assembled with the CLC Genomics Workbench version 6.5.1, and then the contigs were mapped to chromosomes 1 and 2 of *V. parahaemolyticus* RIMD 2210633 using the CONTIGuator program (4). The sequence data were searched for homologous sequences among the strains with the BLASTn program (5).

The genomes of *V. parahaemolyticus* strains TUMSAT_DE1_S1, TUMSAT_DE2_S2, TUMSAT_D06_S3, TUMSAT_H01_S4, TUMSAT_H03_S5, and TUMSAT_H10_S6 were sequenced and assembled into 127, 96, 69, 70, 59, and 64 contigs, respectively. Although a number of the contigs were mapped onto the genome sequences of *V. parahaemolyticus* RIMD 2210633 chromosomes 1 and 2, all of the strains lack the 140-kbp region in chromosome 2 (1,387,700 to 1,467,700) that contains the pathogenicity island. The region encodes a type III secretion system and is considered to be a pathogenic island of the reference strain (6). Another type III secretion system, which is considered not to be pathogenic to humans, has been identified on chromosome 1. This region was found on chromosome 1 of each of the 6 strains in this study.

A comparison of the contigs showing identity to the chromosomes of *V. parahaemolyticus* RIMD 2210633 did not reveal any sequences specific to the strains. The three AHPND strains had some contigs that were not homologous to the chromosomes of known *V. parahaemolyticus* strains. However, each of these contigs was homologous to contigs from the other AHPND strains. For example, contig 4 of TUMSAT_D06_S3 (63 kbp) was highly conserved in the other AHPND strains but was not conserved in the three non-AHPND strains. Contig 4 encodes the homologues of type IV pilus proteins and conjugal transfer proteins, which suggests that it is located on a plasmid.

Here, we sequenced 6 strains of *V. parahaemolyticus*, 3 from diseased shrimps and the others from different shrimp ponds. The pathogenic strains possess conserved sequences that so far have not been reported in other strains of *V. parahaemolyticus*. Further characterization of the sequences may reveal the pathogenesis of the AHPND strains.

### Nucleotide sequence accession numbers.

The genome sequences of 6 strains have been deposited in DDBJ/EMBL/GenBank under the accession no. BAVF01000001 to BAVF01000127, BAVG01000001 to BAVG01000096, BAVH01000001 to BAVH01000069, BAVI01000001 to BAVI01000070, BAVJ01000001 to BAVJ01000059, and BAVK01000001 to BAVK01000064.
